# Association between non-obese diabetes versus obese diabetes on dementia and mortality All of Us research program

**DOI:** 10.1093/geroni/igaf122.3542

**Published:** 2025-12-31

**Authors:** Junyu Sui, Bei Wu, Ross Andel, Xiang Qi

**Affiliations:** Arizona State University, Phoenix, Arizona, United States; NYU Shanghai, China (People’s Republic); Arizona State University, Phoenix, Arizona, United States; New York University, New York, New York, United States

## Abstract

Diabetes mellitus represents a significant risk factor for dementia, with both Type 1 and Type 2 diabetes contributing to cognitive decline through distinct mechanisms including insulin resistance, tau hyperphosphorylation, β-amyloid accumulation, chronic inflammation, and oxidative stress. To compare dementia risk between non-obese and obese individuals with type 1 diabetes (T1D) and type 2 diabetes (T2D) among US adults aged over 50 years, this retrospective study utilized data from the All of Us research program and ICD-9/10 codes to identify T1D, T2D, and dementia cases. Participants were classified as non-obese based on BMI (< 30 kg/m² for non-Asian races; < 27.5 kg/m² for Asian Americans) and waist circumference (< 102 cm for males, < 88 cm for females). Statistical analyses employed Cox-proportional hazard models and competing risk models accounting for mortality. Among 268,648 participants with a median follow-up of 10 (interquartile range, 6–14) years, 216 (17.8%) participants developed dementia. After full adjustment, non-obese T1D demonstrated a 4 times higher risk of dementia (95% CI: 2.86-5.60), while non-obese T2D showed a 1.82 times higher risk (95% CI: 1.45-2.28) compared to those without diabetes. These associations persisted when accounting for competing mortality risk and when using waist circumference as an obesity measure. Non-obese diabetes, particularly T1D, is associated with a significantly greater dementia risk compared to both obese diabetes and no diabetes. These findings underscore the importance of early cognitive screening, careful glycemic management, and targeted prevention strategies for non-obese individuals with diabetes.

